# Supplementary figures and images for: Association between proton pump inhibitor use and upper gastrointestinal cancer: A matched case-control study accounting for reverse causation and confounding by indication
Source: PLoS Med. 2026 Jan 6;23(1):e1004842. doi: 10.1371/journal.pmed.1004842 (PMC12773814; doi:10.1371/journal.pmed.1004842)

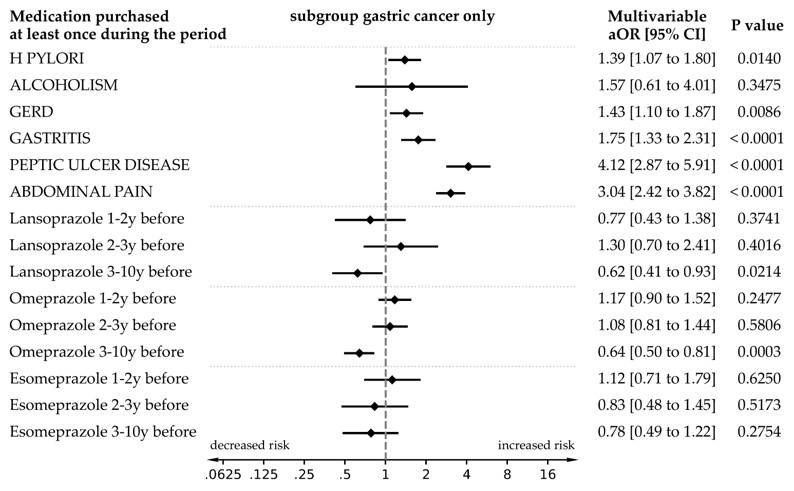

Supplement: S1 Fig — Forest plot of adjusted odds ratios (aORs) for PPI use and registry-confirmed gastric cancer (701 cases; 7,010 matched controls), by exposure windows (1–2 years, 2–3 years, 3–10 years before diagnosis). Estimates from conditional logistic regression within 1:10 matched sets; models adjust for age, smoking, alcohol use, BMI category, socioeconomic status, healthcare utilization, pregnancy history (women), H. pylori diagnosis, and upper GI symptom-related diagnoses. Error bars indicate 95% confidence intervals (CIs). (JPG) [file pmed.1004842.s002.jpg]

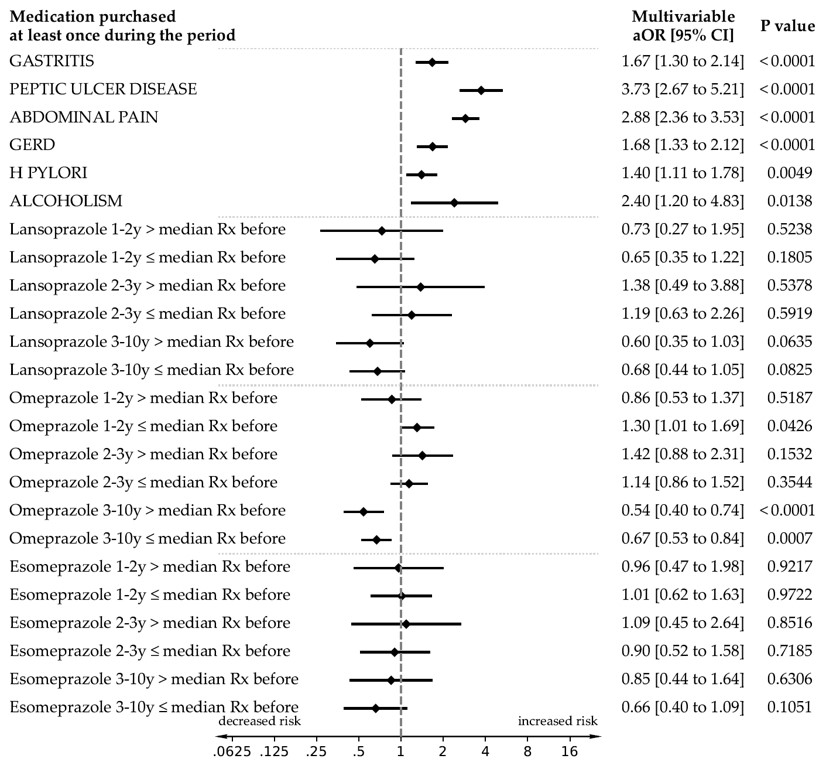

Supplement: S2 Fig — Forest plot comparing higher versus lower cumulative prescription counts within each exposure window (median split) for each PPI. Models and adjustments as in S1 Fig.; conditional logistic regression within matched sets. Error bars show 95% CIs. (JPG) [file pmed.1004842.s003.jpg]
