# Supplementary material for: Association between proton pump inhibitor use and upper gastrointestinal cancer: A matched case-control study accounting for reverse causation and confounding by indication
Source: PLoS Med. 2026 Jan 6;23(1):e1004842. doi: 10.1371/journal.pmed.1004842 (PMC12773814; doi:10.1371/journal.pmed.1004842)
Supplement: Attachment — Submitted filename: Review.docx [file pmed.1004842.s011.docx]

Review on: Association between Proton Pump Inhibitor Use and Upper Gastrointestinal Cancer: A Matched Case-Control Study Accounting for Reverse Causation and Confounding by Indication

This study leveraged national Israeli electronic health records in a matched case-control design to evaluate the association between PPI use and upper gastrointestinal (GI) cancer, explicitly addressing confounding by indication and reverse causation. Initial analyses suggested increased risk with Proton Pump Inhibitor (PPI) use in the five years before diagnosis, but the signal was concentrated in the six months preceding diagnosis; after excluding the final year and adjusting for symptom-related diagnoses, the association disappeared. More remote PPI exposure was associated with lower risk. Overall, the findings indicate that previously reported harmful associations are largely driven by potential bias.

The authors address a timely and controversial topic regarding PPI use. By stratifying exposure across different time windows, they aim to demonstrate that the observed association between PPI use and upper GI cancers is primarily driven by confounding by indication and reverse causation. While this overarching conclusion is plausible, the current analyses do not yet provide sufficiently robust support. The manuscript would be strengthened by the following:

1. Insufficient covariate adjustment. Beyond the variables included, several established risk factors for upper GI cancers were not accounted for, such as dietary patterns, family history, alcohol consumption, and overall comorbidity burden. Please consider incorporating these variables where possible and conducting sensitivity analyses to mitigate residual confounding.
2. Missing dose–response assessment. Exposure was defined as >= 1 pharmacy purchase within a given time window, which may conflate occasional and long-term users. Consider incorporating measures of cumulative exposure (e.g., total prescription days). Prior studies commonly examine dose metrics in relation to upper GI cancer incidence. To more comprehensively argue against a harmful association, please include dose–response analyses, including tests for threshold effects and potential nonlinearity.
3. Insufficient outcome stratification. The Introduction emphasizes prior work on PPIs and gastric cancer. To align with this framing and enhance specificity, please stratify outcomes by anatomic site and report site-specific estimates.
4. Limited description of the matching strategy. Please justify the choice of the 1:10 matching ratio and report post-match balance diagnostics and corresponding statistical handling.
5. Abbreviation consistency. Provide full terms at first mention only and use standardized abbreviations consistently thereafter.
6. Study design extensions. To further address the possibility of a causal effect of long-term PPI use, consider Mendelian randomization analyses, using genetic variants as instrumental variables where feasible.
7. External validity. The study population is limited to Israel, whereas prior literature is largely from Europe, North America, and Asia. To improve generalizability, consider validating findings in additional regions; if not feasible, expand the discussion of this limitation.

Overall, while the research topic is timely and valuable, the current analysis is insufficient and does not meet the PLOS Medicine's publication standards. For this reason, I recommend that this manuscript not be accepted for publication. I hope these comments will help the author team improve the quality of the manuscript.
