# Supplementary material for: Association between proton pump inhibitor use and upper gastrointestinal cancer: A matched case-control study accounting for reverse causation and confounding by indication
Source: PLoS Med. 2026 Jan 6;23(1):e1004842. doi: 10.1371/journal.pmed.1004842 (PMC12773814; doi:10.1371/journal.pmed.1004842)
Supplement: Attachment — Submitted filename: PPI response.docx [file pmed.1004842.s013.docx]

**Comments from the academic editor:**

This is a timely and important topic, as a wide body of research has implicated PPIs as potential risk factors for a variety of conditions. In this well-designed and carefully analyzed study, the authors demonstrate that the initially observed risk was explained by underlying methodological biases, including protopathic bias. The findings are clinically relevant and advance the field methodologically.

The reviewer comments are insightful and constructive. I agree with the recommendations, particularly those from the first reviewer who highlighted important considerations for strengthening the analysis. I also agree that generalizability to other populations, such as Asian countries with a higher incidence of gastric cancer, is a limitation. This could be addressed more explicitly in the discussion.

**Response:**
We thank the Academic Editor for the positive evaluation of our manuscript and for highlighting the importance of our study in advancing methodological rigor in this area. We are pleased that the editor found the study to be well-designed and clinically relevant.

In accordance with the suggestion to more explicitly address the generalizability of our findings, we have revised the Discussion to include a dedicated paragraph acknowledging this limitation. (lines 412–416) and conclusion block (lines 417–423)

We appreciate the constructive guidance and the opportunity to revise our manuscript accordingly.

**Comments from the reviewers:**

Reviewer #1: Review on: Association between Proton Pump Inhibitor Use and Upper Gastrointestinal Cancer: A Matched Case-Control Study Accounting for Reverse Causation and Confounding by Indication

This study leveraged national Israeli electronic health records in a matched case-control design to evaluate the association between PPI use and upper gastrointestinal (GI) cancer, explicitly addressing confounding by indication and reverse causation. Initial analyses suggested increased risk with Proton Pump Inhibitor (PPI) use in the five years before diagnosis, but the signal was concentrated in the six months preceding diagnosis; after excluding the final year and adjusting for symptom-related diagnoses, the association disappeared. More remote PPI exposure was associated with lower risk. Overall, the findings indicate that previously reported harmful associations are largely driven by potential bias.

**Response:**
We thank the reviewer for their thoughtful summary and for recognizing the methodological strengths of our study, particularly our efforts to address confounding by indication and reverse causation. We are encouraged by the acknowledgment that our findings help clarify prior concerns regarding long-term PPI use and cancer risk.

The authors address a timely and controversial topic regarding PPI use. By stratifying exposure across different time windows, they aim to demonstrate that the observed association between PPI use and upper GI cancers is primarily driven by confounding by indication and reverse causation. While this overarching conclusion is plausible, the current analyses do not yet provide sufficiently robust support. The manuscript would be strengthened by the following:

1.Insufficient covariate adjustment. Beyond the variables included, several established risk factors for upper GI cancers were not accounted for, such as dietary patterns, family history, alcohol consumption, and overall comorbidity burden. Please consider incorporating these variables where possible and conducting sensitivity analyses to mitigate residual confounding.

**Response:**
We thank the reviewer for this important suggestion. In response, we have strengthened our covariate adjustment by incorporating additional risk factors extractable from our electronic health record (EHR) system. Specifically, we now adjust for the following variables in our multivariable conditional logistic regression models:

- **Demographics**: age, sex
- **Lifestyle**: smoking status (non-smoker [reference], past smoker, current smoker), alcohol consumption
- **Socioeconomic status (SES)**: categorical variable based on a national 1–20 scale, grouped into 1–3 (reference), 4–6, 7–9, 10–11, 12–14, and 15+, with an additional category for missing SES
- **Healthcare utilization**: number of physician visits in the year before index date
- **BMI**: treated as a categorical variable with 18.5–25 kg/m² as the reference category; other ranges include:
  10–18.5, 25–30, 30–35, 35–40, 40–45, 45–50, 50–55, 55–67
- **Other factors**: healthcare worker status, pregnancy history (in women)

These additional adjustments did not materially alter our main findings.

Although we lacked direct data on dietary patterns, we note that body mass index (BMI)—which was included as a covariate—serves as an objective, cumulative proxy for nutritional intake. Unlike dietary questionnaires, which are prone to recall bias and misclassification, BMI, which is well documented in our health organization, offers a reproducible and clinically relevant indicator of energy balance and dietary behavior over time.

We have updated both the Methods (added Covariates section) and Discussion to reflect these changes and acknowledge the limitations related to unmeasured confounders.

2.Missing dose-response assessment. Exposure was defined as >= 1 pharmacy purchase within a given time window, which may conflate occasional and long-term users. Consider incorporating measures of cumulative exposure (e.g., total prescription days). Prior studies commonly examine dose metrics in relation to upper GI cancer incidence. To more comprehensively argue against a harmful association, please include dose-response analyses, including tests for threshold effects and potential nonlinearity.

**Response:**
We thank the reviewer for this important suggestion. In response, we conducted a sensitivity analysis to evaluate potential dose-response relationships. For each medication exposure variable, we have stratified exposure within each time window based on the cumulative number of purchases, dichotomized at the median value observed in the cohort. This allowed us to compare higher versus lower cumulative use while maintaining alignment with the time-resolved structure of our primary models.

This additional analysis (Supplementary Figure 2) confirmed the same temporal patterns as the main analysis. Specifically, we observed no harmful association for high cumulative exposure when restricting to remote use (i.e., >1 year before diagnosis) and adjusting for underlying gastrointestinal diagnoses.

Moreover, in the 3–10 year window, the protective association observed for omeprazole and lansoprazole was more pronounced in the higher-exposure group—for example, omeprazole aOR 0.54 (95% CI 0.40 to 0.74; p < 0.0001) for high users versus aOR 0.67 (95% CI 0.53 to 0.84; p = 0.0007) for low users, consistent with a possible dose-dependent protective effect. However, this stratification also reduced statistical power, and some comparisons did not reach significance despite showing similar trends.

These findings further support the conclusion that long-term PPI use does not increase upper GI cancer risk, and may be associated with reduced risk for remote use. The use of dichotomized cumulative exposure was adopted as a pragmatic approach, given limitations in assessing daily medication intake.

See: S2 Fig.: Dose–response (dichotomized) analysis of PPI purchases.
Results section.

3.Insufficient outcome stratification. The Introduction emphasizes prior work on PPIs and gastric cancer. To align with this framing and enhance specificity, please stratify outcomes by anatomic site and report site-specific estimates.

**Response:**
We thank the reviewer for this important comment. All cases included in our study were upper gastrointestinal cancers, the majority (701 of 875),being gastric cancers.

To address the reviewer’s suggestion, we conducted a subgroup analysis restricted to these 701 gastric cancer cases and their 7,010 matched controls (Supplementary Figure S1). The observed patterns were consistent with those of the main analysis: after excluding the final year before diagnosis and adjusting for symptom-related diagnoses, we found no evidence of increased cancer risk associated with PPI use. Furthermore, remote PPI use (3–10 years prior to diagnosis) was associated with decreased gastric cancer odds, particularly for lansoprazole and omeprazole. These findings suggest that the absence of harmful association extends specifically to gastric cancer and further strengthen the robustness and generalizability of our conclusions.

See: S1 Fig.: Subgroup analysis: Gastric cancer only

4.Limited description of the matching strategy. Please justify the choice of the 1:10 matching ratio and report post-match balance diagnostics and corresponding statistical handling.

**Response:**
We thank the reviewer for this helpful comment. We have now expanded the description of our matching strategy in the Methods section. Specifically, we clarify that a 1:10 case-to-control matching ratio was selected to increase statistical power and precision of estimated odds ratios, given the relative rarity of upper GI cancers in our dataset and the availability of a large eligible control pool. This ratio allowed for more efficient estimation without introducing substantial variance inflation.

We also added a description of the balance diagnostics performed post-matching. We evaluated covariate balance between cases and matched controls on key baseline variables (e.g., age, sex, ethnic group, smoking status, alcohol-related diagnoses, BMI) using standardized mean differences (SMD) and p-values. These diagnostics confirmed that matched baseline characteristics were well-balanced across matched groups.

See: Methods “Study Population and Matching, and balance statement in Results Table 1 narrative; Table 1 legend and footnote show SMDs and global p-values; matched variables set to N/A (sex, ethnic group)

5.Abbreviation consistency. Provide full terms at first mention only and use standardized abbreviations consistently thereafter.

**Response:**

We thank the reviewer for this helpful suggestion. We have carefully reviewed the manuscript to ensure that all abbreviations are introduced in full at first mention and used consistently thereafter. Additionally, we have included a list of abbreviations (in alphabetical order) at the end of the manuscript for clarity

6.Study design extensions. To further address the possibility of a causal effect of long-term PPI use, consider Mendelian randomization analyses, using genetic variants as instrumental variables where feasible.

**Response:**
We thank the reviewer for this thoughtful suggestion. However, our database does not include genotyping or genome-wide association data, which precludes the use of Mendelian randomization analyses in the current study. We agree that such analyses would be valuable for strengthening causal inference and encourage future research using linked genetic datasets to further investigate this question. We have now added this point as a limitation in the Discussion. (lines 409-412)

7.External validity. The study population is limited to Israel, whereas prior literature is largely from Europe, North America, and Asia. To improve generalizability, consider validating findings in additional regions; if not feasible, expand the discussion of this limitation.

**Response:**
We thank the reviewer for this comment. We agree that validation in other regions would enhance the generalizability of our findings. Unfortunately, we do not currently have access to comparable datasets from Europe, North America, or Asia. We have expanded the Discussion to acknowledge this limitation: (lines 412–416) and conclusion block (lines 417–423)

Overall, while the research topic is timely and valuable, the current analysis is insufficient and does not meet the PLOS Medicine's publication standards. For this reason, I recommend that this manuscript not be accepted for publication. I hope these comments will help the author team improve the quality of the manuscript.

**Response:**

We thank the reviewer for their meticulous assessment of our work. We have carefully addressed all specific comments and substantially revised the manuscript to improve the clarity, methodological rigor, and completeness of the analyses. We believe that these revisions now meet the standards of *PLOS Medicine* and strengthen the overall contribution of our study.

Reviewer #2: In general, this is a well-conducted retrospective study that aims to address whether previously reported associations between receipt of PPIs and subsequent GI cancer might be due to reverse causation. Two strengths are the data source and the careful consideration of straightforward, but appropriate statistical analyses. My comments are only minor.

**Response:**

We thank the reviewer for their positive evaluation of our study and for recognizing the strengths of our data source and analytic approach. We appreciate the constructive minor comments provided and have addressed them carefully in the revised manuscript.

First, it wasn't exactly clear what variables were used for the matching. For instance, the matching section (page 8) implies that socioeconomic status was used for matching, but the figures/tables implied that socioeconomic status was not used for matching, and that furthermore, age /was/ used for matching. Be clear which matching variables were used; for fuzzy or imperfect matches, also provide some quantification regarding the degree to which the controls may have differed from the cases.

**Response:**

We thank the reviewer for pointing this out. We have clarified the matching variables and the handling of age in the Methods. Specifically, cases were matched 1:10 to controls on sex, ethnic sector (general population, Ultra-Orthodox Jewish, or Arab), socioeconomic status (SES), and year of enrollment in Leumit Health Services. Age was not an exact matching key, but for each case we selected controls with the nearest date of birth, which yielded virtually identical age distributions. Post-match balance diagnostics are provided in Table 1 (with p-values now marked “N/A” for exactly matched variables).

Age was nearly identical in cases and controls (63.0 years, SD 11.9). Non-matched variables that showed residual differences (e.g., BMI and selected clinical/lifestyle covariates) were adjusted for in the multivariable models.

See: Matching description: Methods “Study Population and Matching” (nearest DOB)

; balance statement in Results Table 1 narrative

Regarding Table 1, for any variables that were matched on, by design they will have the same distribution between the cases and controls; it may be worth simply having N/A or - or some such in the p-value column since it is not worth describing potential differences between them. Furthermore, for the categorical variables with multiple categories, I found it very strange that a Fisher's Exact Test was used for each of the various levels instead of the entire variable at once (e.g., a single p-value looking at associations between BMI category and case/control status, instead of the four group-specific p-values). You are simply looking for an association between a four-level categorical variable (BMI category) and a binary variable (case/control). There should only be a single p-value.

**Response:**

We thank the reviewer for this comment, and have revised table 1. In the revised table, variables used for matching case and controls have been labelled N/A*, and the legend below table states: “*Not applicable. This variable was used to match case and controls”. These variables include: sex, and ethnicity. Parameters not used for matching case and control have retained their p-values.

For the multivariable models, I didn't find explicitly what the non-exposure variables that were controlled for were. Are they the variables listed in the "Covariates" subsection of the methods? If so, I found it a bit odd that you did not adjust for some variables which were found to have been statistically significantly different between the cases and controls (e.g., BMI category). Please be explicit regarding which variables were in each of your multivariable logistic regression models, and consider including a more comprehensive set of variables to control for.

**Response:**
We have improved our adjustment of the adjusted odds ratios (aORs) and have notably added BMI category as covariate in conditional logistic regression within matched sets.

All models are now adjusted for: sex (matching factor), ethnic sector (general population, Ultra-Orthodox Jewish, Arab; matching factor), socioeconomic status (SES; matching factor), year of enrollment (matching factor), age (continuous; closely matched via nearest date of birth, and residually adjusted in the regression). smoking status (non-smoker, past, current), alcohol use (yes/no), BMI category, healthcare utilization (number of physician visits in the prior 5 years, continuous), pregnancy history (women only), and healthcare-worker status.

In addition, models adjusted for upper GI symptom-related diagnoses include symptoms/diseases recorded before the index date (e.g., gastroesophageal reflux disease, dyspepsia, gastritis, peptic ulcer disease) and *Helicobacter pylori* diagnosis. (ICD-9 code sets and counts provided in Supplementary Tables.)

See: "Covariates” subsection and Figure legends.

Finally, there is a bit of a misstatement in the conclusion: "When appropriately adjusted for diagnostic context and timing, PPI use is not associated with increased cancer risk and may be associated with lower risk when used in the more distant past." implies that there is no association. However, absence of evidence is not evidence of absence; it is more appropriate to conclude that you failed to find an association between these two variables after adjusting for diagnostic context and timing, not that affirmatively no such association exists.

**Response:**
We thank the reviewer for this comment. We have revised the conclusion to avoid over-interpretation and to distinguish between lack of evidence for harm and the absence of any harm.

Reviewer #3: The authors underestimates the previous concerns regarding use of PPI regarding gastric cancer by only referring to one paper on PPI use in older adults.

**Response:**
Thank you for the suggestion. We expanded the Introduction to more fully acknowledge prior concerns, including animal data, mechanistic pathways, and recent meta-analytic syntheses. We now cite eight distinct references regarding PPI use and gastric cancer risk (Abrahami et al., 2022; Cheung and Leung, 2019; Joo et al., 2019; Ko et al., 2016; Peng et al., 2023; Segna et al., 2021; Waldum et al., 2018; Xia et al., 2024; Zhang et al., 2025), including a recent meta-analysis (Zhang et al., 2025).

In the discussion it should be referred to the long-term studies in the rats in the middle of the eighties describing neuroendocrine tumors.

**Response:**
Thank you. We added the historical long-term rat data showing ECL-cell neuroendocrine tumors with sustained acid suppression (Havu, 1986), along with more recent pathology literature discussing ECL hyperplasia and NETs in the context of acid inhibition.

Similar finding with the insurmountable histamine-2 blocker loxtidine so it was evident that the tumors were due to profound acid inhibition leading to the trophic effect of gastrin on its target cell, the ECL cell.

**Response:**
We agree that trophic effects mediated by hypergastrinemia have been implicated with potent acid suppression, including with H2RBs such as loxtidine, and we have cited a relevant review summarizing gastrin levels and gastric histology under long-term acid suppression (Lundell et al., 2015).

Later it has been shown that every condition  with long-term hypergastrinemia in man and animals predisposes to gastric cancer.

**Response:**
Thank you for the suggestion. We added a reference discussing the role of gastrin in gastric carcinogenesis in both man and animal (Smith et al., 2017) to acknowledge this mechanistic line of evidence.

Even the carcinogenic effect of H. pylori infection may be due to hypergastrinemia secondary to hypergastrinemia (Scand J Gastroenterol. 2025 Jul;60(7):652-663. doi: 10.1080/00365521.2025.2509094. Epub 2025 May 24.PMID: 40411354 ). H. pylori infection most often in childhood, and gastric cancer is mainly a disease of old age.

Accordingly, the observation period of 10 years as in the present study is far too short.

**Response:**
We have added a reference to (Waldum et al., 2018). We agree that latency may exceed 10 years, particularly for pathways initiated in childhood infection. Our study was constrained by EHR availability over the preceding decade. We now acknowledge that this window may not capture all long-latency effects.

Also, there is little reason to include the esophagus and gut in studies related to PPI carcinogenesis (for the gut there is a theoretical possibility that microbiological changes due to PPI could predispose to cancer).

**Response:**
Our study considered upper GI cancers as a group, in line with prior works. We have now also added a gastric-cancer only subgroup analysis, which yielded patterns consistent with the main analysis. (Results, Figure S1)

The relatively short latency of gastric cancer by using profound acid inhibitors after H. pylori eradication indicates additive effect via the same mechanism (hypergastrinemia) (Cheung et al. Gut 2018 ; 67 : 28-35).

**Response:**
We acknowledge the post-eradication concern and cite (Cheung et al., 2018). in the Introduction to situate our work. Regarding dose-response, we have added sensitivity analyses that stratified exposure by cumulative prescriptions within time windows (Figure S2); these did not reveal harmful associations after accounting for diagnostic context and timing.

Hypergastrinemia alone induces  malignant gastric neoplasia after at least two decades (Calvete et al. Hum Mol Genet 2015 24:2914-22).

**Response:**
We thank the reviewer for this comment. We have added the suggested citation (Calvete et al., 2015) to acknowledge evidence that sustained hypergastrinemia can promote malignant gastric neoplasia over long time frames. We also now explicitly note in the Discussion that our 10-year EHR observation window may be insufficient to capture very long-latency pathways, particularly those potentially initiated by childhood *H. pylori* infection.

Furthermore, the H. pylori positivity was relatively low among controls as well as cancer patients. What method was used? The present study confirms that initiating PPIs in relatively old people not H. pylori infected  for some years is relatively safe with respect to gastric cancer. The results must not be transformed to the same conclusion in younger individuals.

**Response:**
We agree that our cohort is predominantly older adults (mean age ~63 years), and we now explicitly note that findings should not be extrapolated to younger populations without further study.

*H. pylori* status was ascertained from the electronic health record using physician-assigned ICD-9 diagnoses recorded prior to the index date. Where available, these entries reflect documentation derived from endoscopy/histology or noninvasive testing (e.g., urea breath test, stool antigen, serology). We included *H. pylori* as a covariate to mitigate confounding of PPI exposure by eradication-related prescribing. As expected, *H. pylori* positivity was associated with higher cancer odds (aOR 1.43; 95% CI 1.13–1.80).

We also clarify that the observed “positivity” reflects documented diagnoses rather than population seroprevalence; thus, EHR-based coding likely under-ascertains lifetime exposure. Published Israeli estimates suggest substantial background prevalence (approximately 45–65% across ethnic groups (MUHSEN et al., 2012)), so the relatively low *H. pylori* diagnosis prevalence in our dataset likely reflects incomplete testing/recording rather than genuinely low prevalence. We have added these clarifications and limitations to the manuscript.

Abrahami D, McDonald EG, Schnitzer ME, Barkun AN, Suissa S, Azoulay L. 2022. Proton pump inhibitors and risk of gastric cancer: population-based cohort study. *Gut* **71**:16–24. doi:10.1136/gutjnl-2021-325097

Calvete O, Reyes J, Zuñiga S, Paumard-Hernández B, Fernández V, Bujanda L, Rodriguez-Pinilla MS, Palacios J, Heine-Suñer D, Banka S, Newman WG, Cañamero M, Pritchard DM, Benítez J. 2015. Exome sequencing identifies ATP4A gene as responsible of an atypical familial type I gastric neuroendocrine tumour. *Hum Mol Genet* **24**:2914–2922. doi:10.1093/hmg/ddv054

Cheung KS, Chan EW, Wong AYS, Chen L, Wong ICK, Leung WK. 2018. Long-term proton pump inhibitors and risk of gastric cancer development after treatment for *Helicobacter pylori* : a population-based study. *Gut* **67**:28–35. doi:10.1136/gutjnl-2017-314605

Cheung KS, Leung WK. 2019. Long-term use of proton-pump inhibitors and risk of gastric cancer: a review of the current evidence. *Therap Adv Gastroenterol* **12**. doi:10.1177/1756284819834511

Havu N. 1986. Enterochromaffin-like cell carcinoids of gastric mucosa in rats after life-long inhibition of gastric secretion. *Digestion* **35 Suppl 1**:42–55. doi:10.1159/000199381

Joo MK, Park J-J, Chun HJ. 2019. Proton pump inhibitor: The dual role in gastric cancer. *World J Gastroenterol* **25**:2058–2070. doi:10.3748/wjg.v25.i17.2058

Ko Y, Tang J, Sanagapalli S, Kim BSM, Leong RW. 2016. Safety of proton pump inhibitors and risk of gastric cancers: review of literature and pathophysiological mechanisms. *Expert Opin Drug Saf* **15**:53–63. doi:10.1517/14740338.2016.1118050

Lundell L, Vieth M, Gibson F, Nagy P, Kahrilas PJ. 2015. Systematic review: the effects of long‐term proton pump inhibitor use on serum gastrin levels and gastric histology. *Aliment Pharmacol Ther* **42**:649–663. doi:10.1111/apt.13324

MUHSEN K, COHEN D, SPUNGIN-BIALIK A, SHOHAT T. 2012. Seroprevalence, correlates and trends of *Helicobacter pylori* infection in the Israeli population. *Epidemiol Infect* **140**:1207–1214. doi:10.1017/S0950268811002081

Peng T-R, Wu T-W, Li C-H. 2023. Association between proton-pump inhibitors and the risk of gastric cancer: a systematic review and meta-analysis. *Int J Clin Oncol* **28**:99–109. doi:10.1007/s10147-022-02253-2

Segna D, Brusselaers N, Glaus D, Krupka N, Misselwitz B. 2021. Association between proton-pump inhibitors and the risk of gastric cancer: a systematic review with meta-analysis. *Therap Adv Gastroenterol* **14**. doi:10.1177/17562848211051463

Smith JP, Nadella S, Osborne N. 2017. Gastrin and Gastric Cancer. *Cell Mol Gastroenterol Hepatol* **4**:75–83. doi:10.1016/j.jcmgh.2017.03.004

Waldum HL, Sørdal Ø, Fossmark R. 2018. Proton pump inhibitors (PPIs) may cause gastric cancer – clinical consequences. *Scand J Gastroenterol* **53**:639–642. doi:10.1080/00365521.2018.1450442

Xia B, He Q, Smith FG, Gkoutos VG, Nirantharakumar K, Kuo ZC, Wang D, Feng Q, Cheung EC, Dai L, Huang J, Yu Y, Meng W, Qin X, Yuan J. 2024. Individualized prevention of proton pump inhibitor related adverse events by risk stratification. *Nat Commun* **15**:3591. doi:10.1038/s41467-024-48007-8

Zhang H, Meng X, Gou D, Liu X, Huang Y, Liu S, Wang H, Li H. 2025. A meta-analysis of the effects of proton pump inhibitors on the risk of gastric cancer. *Medicine* **104**:e43053. doi:10.1097/MD.0000000000043053

---------------------------------------------------------

General editorial requests:

(Note: not all will apply to your paper, but please check each item carefully)

We have implemented all editorial requests.

*       We ask every co-author listed on the manuscript to fill in a contributing author statement, making sure to declare all competing interests. If any of the co-authors have not filled in the statement, we will remind them to do so when the paper is revised. If all statements are not completed in a timely fashion this could hold up the re-review process. If new competing interests are declared later in the revision process, this may also hold up the submission. Should there be a problem getting one of your co-authors to fill in a statement we will be in contact. Please do not add or remove authors without first discussing this with the handling editor. You can see our competing interests policy here: <http://journals.plos.org/plosmedicine/s/competing-interests>.

All co-authors will complete the contributing author statements and competing interests. No authors were added/removed.

*       Please upload any figures associated with your paper as individual TIF or EPS files with 300dpi resolution at resubmission; please read our figure guidelines for more information on our requirements: <http://journals.plos.org/plosmedicine/s/figures>. While revising your submission, we strongly recommend that you use PLOS's NAAS tool (<https://ngplosjournals.pagemajik.ai/artanalysis>) to test your figure files. NAAS can convert your figure files to the TIFF file type and meet basic requirements (such as print size, resolution), or provide you with a report on issues that do not meet our requirements and that NAAS cannot fix.

After uploading your figures to PLOS's NAAS tool - <https://ngplosjournals.pagemajik.ai/artanalysis>, NAAS will process the files provided and display the results in the "Uploaded Files" section of the page as the processing is complete. If the uploaded figures meet our requirements (or NAAS is able to fix the files to meet our requirements), the figure will be marked as "fixed" above. If NAAS is unable to fix the files, a red "failed" label will appear above.

When NAAS has confirmed that the figure files meet our requirements, please download the file via the download option, and include these NAAS processed figure files when submitting your revised manuscript.

We have re-exported each figure as single-page TIFF (sRGB, 8-bit, LZW, 600 dpi) within size and inch limits; validated with PLOS’s NAAS tool and uploaded the NAAS-processed versions. Legends define all abbreviations; color palettes avoid red/green pairings.

*       Please ensure that the paper adheres to the PLOS Data Availability Policy (see <http://journals.plos.org/plosmedicine/s/data-availability>), which requires that all data underlying the study's findings be provided in a repository or as Supporting Information. For data residing with a third party, authors are required to provide instructions with contact information (web or email address) for obtaining the data. Please note that a study author cannot be the contact person for the data. PLOS journals do not allow statements supported by "data not shown" or "unpublished results." For such statements, authors must provide supporting data or cite public sources that include it.

We have revised Data Availability Statement to specify the restriction (individual-level clinical data), IRB oversight, and a **non-author contact** for access. (line 465-473):

*       We expect all researchers with submissions to PLOS in which author-generated code underpins the findings in the manuscript to make all author-generated code available without restrictions upon publication of the work. In cases where code is central to the manuscript, we may require the code to be made available as a condition of publication. Authors are responsible for ensuring that the code is reusable and well documented. Please make any custom code available, either as part of your data deposition or as a supplementary file. Please add a sentence to your data availability statement regarding any code used in the study, e.g. "The code used in the analysis is available from Github [URL] and archived in Zenodo [DOI link]" Please review our guidelines at <https://journals.plos.org/plosmedicine/s/materials-software-and-code-sharing> and ensure that your code is shared in a way that follows best practice and facilitates reproducibility and reuse. Because Github depositions can be readily changed or deleted, we encourage you to make a permanent DOI'd copy (e.g. in Zenodo) and provide the URL.

Source code sharing: The statistical analysis was performed using R. The code used in the analysis is available from Github https://github.com/arielisr/upper_gi_models and archived in Zenodo 10.5281/zenodo.17155577

*       The Data Availability Statement (DAS) requires revision: If the data are not freely available, please describe briefly the ethical, legal, or contractual restriction that prevents you from sharing it. Please also include an appropriate contact (web or email address) for inquiries (this cannot be a study author).

Data Availability Statement: “Data sharing: The individual-level electronic health record data underlying this study contain potentially identifiable health information and cannot be shared publicly due to ethical, legal, and contractual restrictions imposed by data-protection regulations in Israel. De-identified data may be made available to qualified researchers upon reasonable request, contingent on approval by the Leumit Health Services Institutional Review Board (IRB) and execution of a data-use agreement. Requests for access should be directed to the Leumit Health Services IRB Secretariat (apincu@leumit.co.il). The study authors are not members of the data access committee and do not have special access privileges; external researchers will be granted access on the same terms following the IRB’s review.”

*       Please include the statement on ethical approval in the Methods section of the main text.

We have included an ethical approval statement in the methos (lines 277-281)

**Ethical approval**

This study was approved by the Leumit Health Services Institutional Review Board (IRB) with a waiver of informed consent (approval number: LEU-0010-21). The waiver was justified on the basis that this large retrospective study used de-identified clinical data analyzed anonymously.

FORMATTING – GENERAL

*       Abstract: Please structure your abstract using the PLOS Medicine headings (Background, Methods and Findings, Conclusions). Please combine the Methods and Findings sections into one section.

Abstract has been reformatted per PLOS headings (Background; Methods and Findings; Conclusions) and combined Methods/Findings; added design, population/setting, sample size, study years, and outcome measures; reported 95% CIs and p-values to journal style.

*       At this stage, we ask that you include a short, non-technical Author Summary of your research to make findings accessible to a wide audience that includes both scientists and non-scientists. The Author Summary should immediately follow the Abstract in your revised manuscript. This text is subject to editorial change and should be distinct from the scientific abstract. Ideally each sub-heading should contain 2-3 single sentence, concise bullet points containing the most salient points from your study. In the final bullet point of 'What Do These Findings Mean?', please include the main limitations of the study in non-technical language. Please see our author guidelines for more information: <https://journals.plos.org/plosmedicine/s/revising-your-manuscript#loc-author-summary>.

We have added a non-technical Author Summary (three sub-headings with concise bullet points); final bullet lists limitations in plain language immediately after Abstract.

*       Please express the main results with 95% CIs as well as p values. When reporting p values please report as p<0.001 and where higher as the exact p value p=0.002, for example. Throughout, suggest reporting statistical information as follows to improve clarity for the reader "22% (95% CI [13%,28%]; p</=)". Please be sure to define all numerical values at first use.

Ensured main results include aORs with 95% CIs and p-values (exact p where ≥0.001; “p<0.001” otherwise). Clarified numerical units/definitions at first use.

*       Please include page numbers and line numbers in the manuscript file. Use continuous line numbers (do not restart the numbering on each page).

We have added page numbers and continuous line numbering.

*       Please cite the reference numbers in square brackets. Citations should precede punctuation.

done

FIGURES AND TABLES

*       Please provide titles and legends for all figures and tables (including those in Supporting Information files).

We have provided complete titles/legends for all tables/figures, including Supporting Information;

See Table and Figure Legends section page 25

*       Please define all abbreviations used in each figure/table (including those in Supporting Information files).

Expanded legends to define all abbreviations used in figure/tables.

*       Please consider avoiding the use of red and green in order to make your figure more accessible to those with color blindness.

SUPPLEMENTARY MATERIAL

*       Please note that supplementary material will be posted as supplied by the authors. Therefore, please amend it according to the relevant comments outlined here.

Done

* Please cite your Supporting Information as outlined here: <https://journals.plos.org/plosmedicine/s/supporting-information>

Done

REFERENCES
*       PLOS uses the numbered citation (citation-sequence) method and first six authors, et al.

Done

*       Please ensure that journal name abbreviations match those found in the National Center for Biotechnology Information (NCBI) databases (<http://www.ncbi.nlm.nih.gov/nlmcatalog/journals>), and are appropriately formatted and capitalised.

Done

*       Where website addresses are cited, please include the complete URL and specify the date of access (e.g. [accessed: 12/06/2023]).

Done

STUDY TYPE-SPECIFIC REQUESTS

*       Abstract: Please include the study design, population and setting, number of participants, years during which the study took place (enrollment and follow up), length of follow up, and main outcome measures.

Done

*       Please ensure that the study is reported according to the STROBE (or appropriate STOBE extension) guideline (available from: <https://www.equator-network.org/reporting-guidelines/strobe>) and include the completed STROBE (or STROBE extension) checklist as Supporting Information. Please add the following statement, or similar, to the Methods: "This study is reported as per the Strengthening the Reporting of Observational Studies in Epidemiology (STROBE) guideline (S1 Checklist)." When completing the checklist, please use section and paragraph numbers, rather than page numbers.

Done

*       For all observational studies, in the manuscript text, please indicate: (1) the specific hypotheses you intended to test, (2) the analytical methods by which you planned to test them, (3) the analyses you actually performed, and (4) when reported analyses differ from those that were planned, transparent explanations for differences that affect the reliability of the study's results. If a reported analysis was performed based on an interesting but unanticipated pattern in the data, please be clear that the analysis was data driven.

Clarified (1) prespecified hypotheses, (2) planned analytical methods, (3) analyses actually performed, and (4) data-driven additions with rationale (dose stratification, gastric-only subgroup) in a dedicated transparency note.

Location: Methods → Protocol and Analysis Plan / Statistical Analyses; Discussion → Limitations/Interpretation.

*       Please state in the Methods section whether the study had a prospective protocol or analysis plan. If a prospective analysis plan (from your funding proposal, IRB or other ethics committee submission, study protocol, or other planning document written before analyzing the data) was used in designing the study, please include the relevant document(s) with your revised manuscript as a Supporting Information file to be published alongside your study and cite it in the Methods section. A legend for this file should be included at the end of your manuscript. If no such document exists, please make sure that the Methods section transparently describes when analyses were planned, and when/why any data-driven changes to analyses took place. Changes in the analysis, including those made in response to peer review comments, should be identified as such in the Methods section of the paper, with rationale

Stated that no prospectively registered analysis plan existed; described when analyses were planned and what changes occurred in response to peer review, with rationale.
