# Supplementary material for: Association between proton pump inhibitor use and upper gastrointestinal cancer: A matched case-control study accounting for reverse causation and confounding by indication
Source: PLoS Med. 2026 Jan 6;23(1):e1004842. doi: 10.1371/journal.pmed.1004842 (PMC12773814; doi:10.1371/journal.pmed.1004842)
Supplement: Attachment — Submitted filename: PPI point-to-point v3.docx [file pmed.1004842.s014.docx]

**Reviewer 1**

*Comment:* The inclusion of dietary data should not only focus on nutrient intake but also on dietary preferences. The explanation in the limitations seems unconvincing.
 *Response:* We clarified this limitation and justified our approach. BMI and SES are recognized proxies for nutritional exposure in Israeli and comparable populations.
**Added text (Discussion, Limitations):**

“Although we lacked direct measures of dietary preferences or family history, BMI serves as a validated proxy for long-term nutritional status and energy intake in Israeli and other populations[41,42]. Detailed dietary preferences were not recorded in the EHR; however, body-mass index and socioeconomic indicators remain the most feasible proxies for cumulative nutritional exposure in this setting. We have therefore made the best possible attempt, within the constraints of our health system, to mitigate dietary confounding. These measures may even provide greater objectivity than dietary questionnaires used in other studies, which are prone to recall and reporting bias.”

*Comment:* This study relies on ICD-9 codes for disease diagnoses. Given that ICD-10 codes are more widely used in current clinical practice, it would be helpful to clarify whether the database used lacks ICD-10 codes. If the dataset only includes ICD-9 codes, there is a risk of missing certain diagnoses, which could affect the results. I suggest that this point be addressed in the limitations section.

*Response:* During the study period, ICD-9 was the only nomenclature used in our health system. However, cancers in the national cancer registry were documented according to ICD-10 classification. We now explain this more clearly in the manuscript.

**Added text (Methods – Study Population):**

During the study period, Leumit Health Services used ICD-9 for clinical coding, whereas the Israeli National Cancer Registry applied ICD-10. To ensure complete ascertainment, we merged both sources and used the earliest record from either system as the index diagnosis date. This dual coding minimized potential under-capture of cancer diagnoses. Of the 892 detected upper GI cancer cases, 680 (76%) were identified via linkage to the Israeli National Cancer Registry, which provided structured data on tumor site, morphology, and histological grade (see S2-S4 Tables) in ICD-10 coded form. The remaining cases were identified through validated ICD-9 codes in the LHS medical record system (ICD-9 codes beginning with 150–152; S5 Table).

*Comment:* While I understand that you acknowledge the limitation of not including data from other regions, I believe the results should still be interpreted with caution. The lack of broader geographic representation may affect the generalizability of your findings, and I suggest that this point be emphasized in the discussion of the implications of the results. Additionally, it would be beneficial to include a statement about the potential for regional differences in disease patterns and outcomes.

*Response:* We have expanded and streamlined the generalizability statement for clarity and balance.

**Revised text (Discussion):**

“Although our cohort was ethnically, culturally, and socioeconomically diverse, regional differences in diet, infection prevalence, and healthcare access could still influence upper gastrointestinal cancer incidence. The generalizability of these findings to populations with different risk profiles—such as those in East Asia, where gastric cancer incidence is higher—remains uncertain. Given the cohort’s age distribution, the results primarily inform older adults and should be extrapolated to younger populations with caution. Replication in other geographic and ethnic settings, including Europe, North America, and Asia, is warranted to confirm external validity”

*Comment:* Previous case-control and cohort studies on PPIs and gastric cancer have shown no statistically significant trend between PPI use and increased gastric cancer risk. It is recommended to add a summary or discussion of relevant articles in the introduction and discussion sections to further clarify the novelty and advantages of the study.

*Response:* This point was already addressed in the Introduction and Discussion through references [32] Moayyedi et al. (Gut, 2019) and [33] Crafa et al. (Acta Biomed, 2023). To respond more explicitly to the reviewer, we added a bridging sentence to reinforce how our study builds upon this prior work.

“Several studies have reported that adjusting for these conditions attenuates or eliminates the association [32], while others have shown that elevated risk estimates cluster shortly before diagnosis, consistent with reverse causation or confounding by indication [33]. Together, these studies suggest that the apparent association between PPI use and gastric cancer observed in some analyses could reflect reverse causation or confounding by indication rather than a true causal relationship. However, the extent to which these biases account for the reported associations remains uncertain, and our study was specifically designed to address this question by modeling exposure timing and diagnostic context.”

**Reviewer 2**

*Comment:* No further comments.

*Response:* We thank the reviewer for the constructive remarks and the final positive evaluation.

**Reviewer 3**

*Comment:* The following aspects should be commented on: The duration of the the PPI use. It should be remembered that the Spanish family with missense of the K+H+-ATPase developed tumors (NETs in the twenties and cancer in the thirties)(Calvete et al). Could the apparent positive effect by the use of PPIs many years before the diagnosis of cancer be due to endoscopy at that time eliminating persons with precancer changes?. Every condition with long-term hypergastrinemia in animals gives gastric neoplasia. Even in man all other causes of long-term hypergastrinemia predisposes to gastric cancer. Eradication of H. pylori followed by PPI treatment causes without doubt gastric cancer and there are many papers describing gastric NETs secondary to PPI treatment, and even some case reports connecting gastric cancer to PPI use. All these aspects should be included in the discussion..

*Response:* We appreciate the reviewer’s detailed mechanistic perspective and have added paragraphs explicitly addressing these points, with appropriate references to Calvete et al. (2015) and related literature.

**Added text (Introduction):**

Several studies, including meta-analyses, have also reported an increased risk of gastric cancer with long-term PPI use [13–21], particularly following *H. pylori* eradication, intensifying debate about whether chronic acid suppression might promote carcinogenesis [22]. Proposed mechanisms center on PPI-induced hypergastrinemia with trophic effects on the gastric mucosa and acid-related shifts in the gastric microbiome leading to mucosal proliferation that may facilitate carcinogenic processes [17,22–26] Consistent with these hypotheses, long-term acid suppression in rats has been associated with enterochromaffin-like (ECL) cell hyperplasia and gastric neuroendocrine tumors [27], and experience with potent H2 receptor blockade supports a gastrin-mediated trophic pathway under chronic hypoacidity [28].

**Added text (Discussion):**

“Persistent hypergastrinemia has been implicated as a trophic stimulus for gastric mucosal proliferation in experimental models[23]. Rare hereditary cases carrying germline ATP4A mutations develop gastric neuroendocrine tumors and adenocarcinoma at young age[40]. However, these conditions represent extreme pathophysiology distinct from routine PPI exposure. Moreover, long-term PPI users in clinical practice undergo more frequent endoscopic evaluation, which could lead to earlier tumor detection (surveillance bias). The lack of excess cancer among such users in our data therefore strengthens the inference that PPIs themselves are unlikely to be causative.”

**Requests from Editors**

**GENERAL**

*** Please confirm that your title complies with to PLOS Medicine's style. Your title must be nondeclarative and not a question. It should begin with main concept if possible. "Effect of" should be used only if causality can be inferred, i.e., for an RCT. Please place the study design ("A randomized controlled trial," "A retrospective study," "A modelling study," etc.) in the subtitle (i.e., after a colon).**

We confirmed that the title is non-declarative and correctly formatted.

*** Statistical reporting: Please revise throughout the manuscript, including tables and figures.**

We have carefully reviewed the statistical reporting throughout the manuscript, including all tables and figures. As requested, we ensured consistent notation of effect measures and confidence intervals, and repeated statistical definitions where appropriate.
With respect to the confidence interval format, we have used the notation “[XX to XX]” consistently throughout the text. This notation has even less potential for ambiguity than commas—avoiding confusion with negative signs, thousands separators, or decimal points—while fully preserving clarity and readability.

*** Please ensure that all abbreviations are defined at first use throughout the text (including statistical abbreviations). Please also check figures and tables.**

All abbreviations are now defined at first use.

*** Please ensure that tables and figures, including those in supplementary files, are appropriately referenced in the main text.**

Tables and figures are cited sequentially and appropriately referenced in the text

*** Please check that any use of statistical terms (such as trend or significant) are supported by the data, and if not please remove them. Please note that the term trend should be used only when the test for trend has been conducted.**

We confirm that all uses of statistical terms are supported by the data. Two instances of the term trend were removed where a formal test for trend had not been conducted.

*** Please review your text for claims of novelty or primacy (e.g. 'for the first time') and remove this language.**

We carefully reviewed the entire manuscript and confirm that it contains no explicit claims of novelty or primacy. No changes were necessary in response to this comment.

*** Your study is observational and therefore causality cannot be inferred. Please remove any language that implies causality, such as effect. Refer to associations instead.**

We agree with the editor's clarification. Our manuscript does not make causal claims; rather, it explicitly refutes prior suggestions of a causal link between PPI use and upper gastrointestinal cancer. We carefully rechecked the text to ensure that all phrasing refers to associations rather than effects, and confirm that no causal language remains.

*** Although "risk" and "odds" are often used interchangeably in common conversation, they have very specific meanings in medical research. Since you are reporting odds ratios, we believe it is clearer to refer to "odds" throughout and remove the word "risk."**

We appreciate this clarification and have carefully reviewed all instances of the term risk throughout the manuscript. We replaced risk with odds wherever it referred to modeled associations derived from odds ratios. However, we retained risk in standard epidemiologic expressions such as risk factor or when citing results or terminology from prior studies that reported risk estimates, to preserve scientific accuracy and contextual fidelity.

*** Please note that you indicated in the online submission form that the study received no funding, yet you provided a financial disclosure statement after the main text. Please revise the online submission form to ensure that all statements are correct and updated according to our requirements.**

We thank the editors for noting this point. The financial disclosure statement clarifies that no external or dedicated grant funding was received for this study. The work was conducted using internal resources of Leumit Health Services, and Dr. Eytan Ruppin’s contribution was supported by the Intramural Research Program of the U.S. National Cancer Institute, which covers his research activities. The online submission form has been updated accordingly to ensure consistency with the following statement included in the manuscript.

*** Please include the statement on code availability in the data availability statement.**

The code availability statement was already present in the manuscript under the heading “Source code sharing.” We have now renamed this section to “Code Availability” for clarity. The statement now reads:

Source code sharing: The statistical analysis was performed using R. The code used in the analysis is available from Github https://github.com/arielisr/upper_gi_models and archived in Zenodo 10.5281/zenodo.17155577

**ABSTRACT**

*** Please confirm that your abstract complies with our requirements, including providing all the information relevant to this study type https://journals.plos.org/plosmedicine/s/submission-guidelines#loc-abstract**

We confirm that the abstract fully complies with PLOS Medicine’s requirements for observational studies. It is structured under Background, Methods and Findings, and Conclusions, includes study design, data source, matching variables, statistical approach, main findings with corresponding adjusted odds ratios and confidence intervals, and appropriately reflects the study’s observational nature without implying causality. No further modifications were necessary.

*** Please clarify what you mean by ‘ethnic sector’. Please also see our comments under 'Methods and Results'.**

We thank the editors for this comment. We clarified the meaning of ethnic sector directly in the abstract and Methods. The abstract now specifies the categories as general, Jewish ultra-Orthodox, and Arab populations, and the Methods section provides further detail on how this classification was derived:

"Matching was performed on age, sex, ethnic sector (general, Jewish ultra-orthodox, Arab), socioeconomic status, and year of enrollment. PPI exposure was ascertained from pharmacy records and modeled in discrete pre-diagnosis windows (0–6 months, 6–12 months, 1–3 years, 3–10 years). Ethnic sector classification was based on residential clustering algorithms validated against census data in prior national studies[1]. The algorithm identifies ethnic sector based on neighborhood demographic composition; ethnicity is not directly recorded in Israeli EHRs. "

*** Please provide the main baseline characteristics of the study population.**

We have added the main baseline characteristics to the abstract to improve clarity. The abstract now specifies:

Cases were 875 adults (age 63.0 ± 11.9, 62.5% male)

*** In the abstract, please confirm that you included the important dependent variables that are adjusted for in the analyses.**

We assume the editors are referring to the key independent variables (covariates) included in the multivariable adjustment. The abstract indeed specifies these variables, namely age, sex, smoking status, socioeconomic status (SES), body-mass index (BMI), and gastrointestinal comorbidities. No further modification was necessary.

*** “we did not detect a harmful association between PPI use and upper GI cancer” – for transparency, please report the numerical values (at least an example).**

We thank the editors for this suggestion. We have added the corresponding numerical examples (aORs and 95% CIs) in the main Results section, where full reporting is appropriate. Including even a single example of a non-significant association, with specification of the medication class, time window, odds ratio, confidence interval, and p-value, would increase the abstract length to approximately 425 words. PLOS Medicine recommends abstracts of around 300 words, although the formal upper limit is 500 words. We believe that further extending the abstract would reduce clarity and readability and would require removal of more critical content. Moreover, making substantive changes to the abstract at this late stage of review, after all three reviewers have evaluated the study based on its current version, would be unadvisable. To preserve clarity and maintain consistency with the reviewed version, we have therefore incorporated these numerical details in the manuscript body but not in the abstract.

Added in Results:

"In a model excluding PPI use during the final year and adjusting for GI diagnoses (Figure 3), no positive association remained between any PPI and cancer odds (e.g. omeprazole 1-2 years before aOR=1.17, 95% CI 0.92 to 1.49, p=0.19). "

**METHODS AND RESULTS**

*** Upon further thought, we believe that the RECORD guideline/checklist might be a better fit for your study. Please ensure that the study is reported according to the RECORD guideline (available from https://www.record-statement.org) and include the completed checklist as Supporting Information. Please add the following statement, or similar, to the Methods: "This study is reported as per the Reporting of Studies Conducted using Observational Routinely-Collected Data (RECORD) guideline (S1 Checklist)." When completing the checklist, please use section and paragraph numbers, rather than page numbers.**

In accordance with the editorial request, we have completed the RECORD checklist using section and paragraph numbers rather than page numbers, as instructed. The statement “This study is reported as per the Reporting of Studies Conducted using Observational Routinely-Collected Data (RECORD) guideline (S1 Checklist)” has been added to the Methods section.

*** “ethnic sector (general population, Ultra-Orthodox Jewish, or Arab) – Could you please clarify how ethnicity/race was recorded and defined? How is the “general population” defined? Is "Ultra-Orthodox Jewish" an officially recognized ethnicity in Israel?**

We thank the editors for this important clarification request. In Israel, ethnicity is not directly recorded in electronic health records. Instead, population sector is routinely inferred from residential clustering patterns validated against national census data. This approach, widely used in Israeli epidemiological studies, classifies neighborhoods as belonging predominantly to one of three demographic sectors: general (mostly non-Ultra-Orthodox Jewish), Ultra-Orthodox Jewish (“Haredi”), or Arab. The “general population” category encompasses all communities not identified as predominantly Ultra-Orthodox or Arab.
This classification has been consistently used in major population-based analyses published in BMJ and NEJM and reflects an accepted convention in Israeli health research. We have clarified this point in the Methods – Study Population.

*** “socioeconomic status (SES), based on residential address and grouped into six ordinal categories” – please provide these categories here.**

We have clarified the SES classification in the *Methods – Covariates, paragraph 3*.

Socioeconomic status was derived from residential address and expressed as a national index ranging from 1 to 20, as defined by the Israeli Central Bureau of Statistics. For analysis, values were grouped into six ordinal categories: 1–3, 4–6, 7–9, 10–11, 12–14, and ≥15. These groupings are consistent with prior national health research and allow for adequate discrimination across the socioeconomic gradient.

*** For BMI, please provide a unit/definition.**

We have clarified the definition of BMI in the *Methods – Covariates, paragraph 3*, as follows:

“Body mass index (BMI, categorical; cutoffs: <18.5, 18.5–25 [reference], 25–30, 30–35, 35–40, 40–45, 45–50, 50–55, ≥55 kg/m²).”
The unit (kg/m²) and category boundaries are now explicitly stated.

*** “Patient and public involvement” – We suggest removing this paragraph, but will leave it up to your discretion.**

The paragraph on patient and public involvement has been removed as suggested.

*** “Reporting guideline” – We suggest adding the statement about the reporting guideline to another section of the Methods, such as "Study Design and Data Source."**

We have added the requested statement at the end of the Methods, Protocol and Analysis Plan section:

This study is reported as per the RECORD (Reporting of Studies Conducted Using Observational Routinely-Collected Data) guideline (S1 Checklist).

*** “Cancer cases” – Please replace ‘cases’ with participant, patient, individual, or person and ensure to use patient-centered language throughout your manuscript. Patient-centered language is constructed with the use of post-modified nouns (e.g. 'patients with cancer’ (or similar) instead of ‘cancer patients’) putting the person first in the sentence structure.**

We have revised the manuscript to use patient-centered language throughout. All instances of “cancer cases” have been replaced with “individuals with cancer” to ensure person-first terminology.

*** Table 2: Please report confidence intervals.**

Confidence intervals have been added for all estimates in Table 2 as requested.

*** l.316, “Famotidine and calcium carbonate were not significantly associated with cancer risk.” – what about Lansoprazole?**

Lansoprazole was already included in the preceding sentence summarizing the PPI-specific results (“ Esomeprazole had the strongest association (adjusted OR 4.01, 95% CI 3.20 to 5.03, p<0.001), followed by omeprazole (aOR 2.38, 95% CI 1.99 to 2.85, p<0.001).”).

The text therefore already addresses this point, and no further change was required.

*** “alcoholism” – Please note that in the Methods section, you refer to alcohol use rather than alcoholism. We suggest using "alcohol use" or "alcohol consumption" throughout.**

We have replaced “alcoholism” with “alcohol use” throughout the manuscript for consistency with the editor's preferred terminology. In our data source, alcohol-related diagnoses refer to documented alcohol use disorders rather than occasional consumption, so the meaning remains unchanged.

*** Figure 3: Since you only have one graph, please note that the six diagnoses are listed under "Medication purchased at least once during the period," which seems inaccurate. We suggest splitting the graph.**

We thank the editors for this observation. We have modified Figure 3 to improve clarity by:

1. Replacing “alcoholism” with “alcohol use,”
2. Updating the title to read “Diagnosis recorded or medication purchased at least once before the index date.”

We did not split the figure, as the analysis is based on a single multivariable model that jointly adjusts for both medication purchases and diagnostic variables. Presenting them together in one panel correctly reflects the structure of the regression model and allows readers to compare their relative effect sizes on a common scale.

*** Table 1: Why aren't alcohol-related diagnoses or alcohol consumption presented in the baseline characteristics?**

We thank the editors for this observation. Alcohol-related diagnoses have now been added to Table 2 under the “Diagnosis recorded” section, alongside other gastrointestinal conditions. Because alcohol-related data are coded as clinical diagnoses rather than continuous exposure measures, they were more appropriately presented in Table 2, which lists all recorded diagnoses within the five years before the index date. The entry “Alcohol use” now appears in the updated table with corresponding case and control counts, odds ratio, and confidence interval.

**DISCUSSION**

*** “In conclusion, our findings do not provide evidence for a causal link between long term PPI use and upper gastrointestinal cancer.” – Please revise with regard to your study being observational.**

**We have revised the sentence to reflect the observational design:**

**“**In conclusion, this observational study does not provide evidence for a causal link between long-term PPI use and upper gastrointestinal cancer**”**

*** It seems that you did not have any data on medication adherence. Please address this as a limitation.**

We have added this limitation to the Discussion – Limitations paragraph:

“Additionally, pharmacy purchase data may not fully capture actual medication adherence, which could introduce nondifferential exposure misclassification.”

**General Editorial Requests

1) We ask every co-author listed on the manuscript to fill in a contributing author statement. If any of the co-authors have not filled in the statement, we will remind them to do so when the paper is revised. If all statements are not completed in a timely fashion this could hold up the re-review process. Should there be a problem getting one of your co-authors to fill in a statement we will be in contact. YOU MUST NOT ADD OR REMOVE AUTHORS UNLESS YOU HAVE ALERTED THE EDITOR HANDLING THE MANUSCRIPT TO THE CHANGE AND THEY SPECIFICALLY HAVE AGREED TO IT.**

All co-authors have completed their individual contributing author statements as required. No changes have been made to the author list.
 **2) Please ensure that the paper adheres to the PLOS Data Availability Policy (see** [**http://journals.plos.org/plosmedicine/s/data-availability**](http://journals.plos.org/plosmedicine/s/data-availability)**), which requires that all data underlying the study's findings be provided in a repository or as Supporting Information. For data residing with a third party, authors are required to provide instructions with contact information for obtaining the data. PLOS journals do not allow statements supported by "data not shown" or "unpublished results." For such statements, authors must provide supporting data or cite public sources that include it.**

The manuscript fully complies with the PLOS Medicine Data Availability Policy. The Data Availability statement specifies that individual-level data cannot be publicly shared due to privacy regulations, but de-identified data can be made available upon IRB-approved request to the Leumit Health Services IRB Secretariat (apincu@leumit.co.il). Code is publicly available on GitHub and archived on Zenodo (doi:10.5281/zenodo.17155577). There is no statement of “data not shown” or “unpublished results” in the manuscript. **3) Please review your reference list to ensure that it is complete and correct. If you have cited papers that have been retracted, please include the rationale for doing so in the manuscript text, or remove these references and replace them with relevant current references. Any changes to the reference list should be mentioned in the rebuttal letter that accompanies your revised manuscript.**

The reference list has been carefully reviewed and verified for completeness and accuracy. No retracted papers are cited.
