# Supplementary material for: Association between proton pump inhibitor use and upper gastrointestinal cancer: A matched case-control study accounting for reverse causation and confounding by indication
Source: PLoS Med. 2026 Jan 6;23(1):e1004842. doi: 10.1371/journal.pmed.1004842 (PMC12773814; doi:10.1371/journal.pmed.1004842)
Supplement: Attachment — Submitted filename: PPI point-to-point v4.docx [file pmed.1004842.s015.docx]

** Title: We suggest changing the title to: Association between Proton Pump Inhibitor Use and Upper Gastrointestinal Cancer: A Matched Case-Control Study*

We respectfully prefer to retain the current title, “Association between Proton Pump Inhibitor Use and Upper Gastrointestinal Cancer: A Matched Case-Control Study Accounting for Reverse Causation and Confounding by Indication.”
This wording accurately reflects the study’s key methodological contribution: the explicit modeling of reverse causation and confounding by indication. This insight, which was emphasized by the reviewers and **is central to the paper’s novelty**.

The current title is compliant with PLOS Medicine’s style guidelines (non-declarative, descriptive, and with study design in the subtitle).

** Abstract: When reporting age, please add ‘year’ as unit.*

**Response:**
Done. The abstract now specifies: “Cases were 875 adults (age 63.0±11.9 years, 62.5% male).”

** Table 2: Please add a heading for the CI values, such as ‘ 95% confidence intervals’.*

**Response:**
Done. Table 2 now includes the heading “95% Confidence Intervals (CI)” above the relevant column.

** Discussion: “In conclusion, this observational study does not provide evidence for a causal link between long-term PPI use and upper gastrointestinal cancer. – Thank you for revising the sentence. Because of the way the study was designed, it would be impossible to show evidence of a causal link, which makes the statement unnecessary. We suggest changing it to: In conclusion, this study does not provide evidence for an association between long-term PPI use and upper gastrointestinal cancer.*
 **Response:**

We believe the conclusion should reflect the key finding of this study.
Our work is not merely another analysis showing no association between long-term PPI use and upper gastrointestinal cancer. Rather, it demonstrates that the previously observed associations between PPI use and upper gastrointestinal cancer—associations we were able to replicate—disappear once diagnostic context and confounding by indication are appropriately accounted for.

This is the central contribution of the study: it shows that the apparent harmful association reported in unadjusted or partially adjusted analyses is largely driven by reverse causation and indication bias. Simplifying the conclusion to state only that “no association was found” would obscure this methodological insight, which is precisely what distinguishes our work from prior observational analyses.

We therefore prefer to use the following concluding sentence, which we think better reflects the insights from our study:

“In conclusion, this observational study demonstrates that the apparent association between long-term PPI use and upper gastrointestinal cancer can be explained by confounding by indication and reverse causation, rather than by a true pharmacologic effect.”

** Thank you addressing our requests regarding the definition of ‘ethnic sector’. In your rebuttal, you have stated that you clarified the meaning of ethnic sector directly in the abstract and Methods. The paragraph you have provided in your response does not reflect the changes in the manuscript and the manuscript is still lacking a reference to the algorithms. Please add the statement from your rebuttal to the Methods Section, “Ethnic sector classification was based on residential clustering algorithms validated against census data in prior national studies[1]. The algorithm identifies ethnic sector based on neighborhood demographic composition; e**thnicity is not directly recorded in Israeli EHRs."*

**Response:**
Done. The statement has been added verbatim to the Methods – Sociodemographic Classification subsection.

** “as defined by the Israeli Central Bureau of Statistics”, “These groupings are consistent with prior national health research and allow for adequate discrimination across the socioeconomic gradient.” – we think it would be useful to add these explanations in the Methods Section.*

**Response:**
Done. The paragraph has been revised as follows:

**Sociodemographic Classification**
Socioeconomic status was derived from residential address using the Points Location Intelligence system, which ranks neighborhoods on a 1–20 scale aligned with the socioeconomic status indicators defined by the Israeli Central Bureau of Statistics. For analysis, values were grouped into six ordinal categories: 1–3, 4–6, 7–9, 10–11, 12–14, and ≥15. These groupings are consistent with prior national health research and allow for adequate discrimination across the socioeconomic gradient.

** Please note that we have updated the data availability statement in the online submission form to include the statement on code availability. We have also updated the Financial Disclosure statement. Please check.*

**Response:**
Confirmed. The updates in the online submission form are correct and consistent with the corresponding statements in the manuscript.

**Summary:**
All editorial comments have been addressed in full, except for the proposed modifications to the title and concluding sentence. Both points are central to the conceptual focus of the study, which demonstrates that the apparent association between PPI use and upper gastrointestinal cancer is driven by confounding by indication and reverse causation rather than by a causal drug effect. All other textual, tabular, and methodological clarifications have been implemented as requested.
